# Supplementary material for: GRB2 stabilizes RAD51 at reversed replication forks suppressing genomic instability and innate immunity against cancer
Source: Nat Commun. 2024 Mar 8;15:2132. doi: 10.1038/s41467-024-46283-y (PMC10923831; doi:10.1038/s41467-024-46283-y)
Supplement: Supplementary file 1 — Supplementary Information [file 41467_2024_46283_MOESM1_ESM.pdf]

GRB2 stabilizes RAD51 at reversed replication forks suppressing genomic instability and innate immunity against cancer.

Zu Ye[1], Shengfeng Xu[2], Yin Shi[3,4], Xueqian Cheng[5], Yuan Zhang[1], Sunetra Roy[6], Sarita Namjoshi[1], Michael A. Longo[1], Todd M. Link[1], Katharina Schlacher[6], Guang Peng[5], Dihua Yu[1], Bin Wang[3], John A. Tainer[1*], Zamal Ahmed[1*]

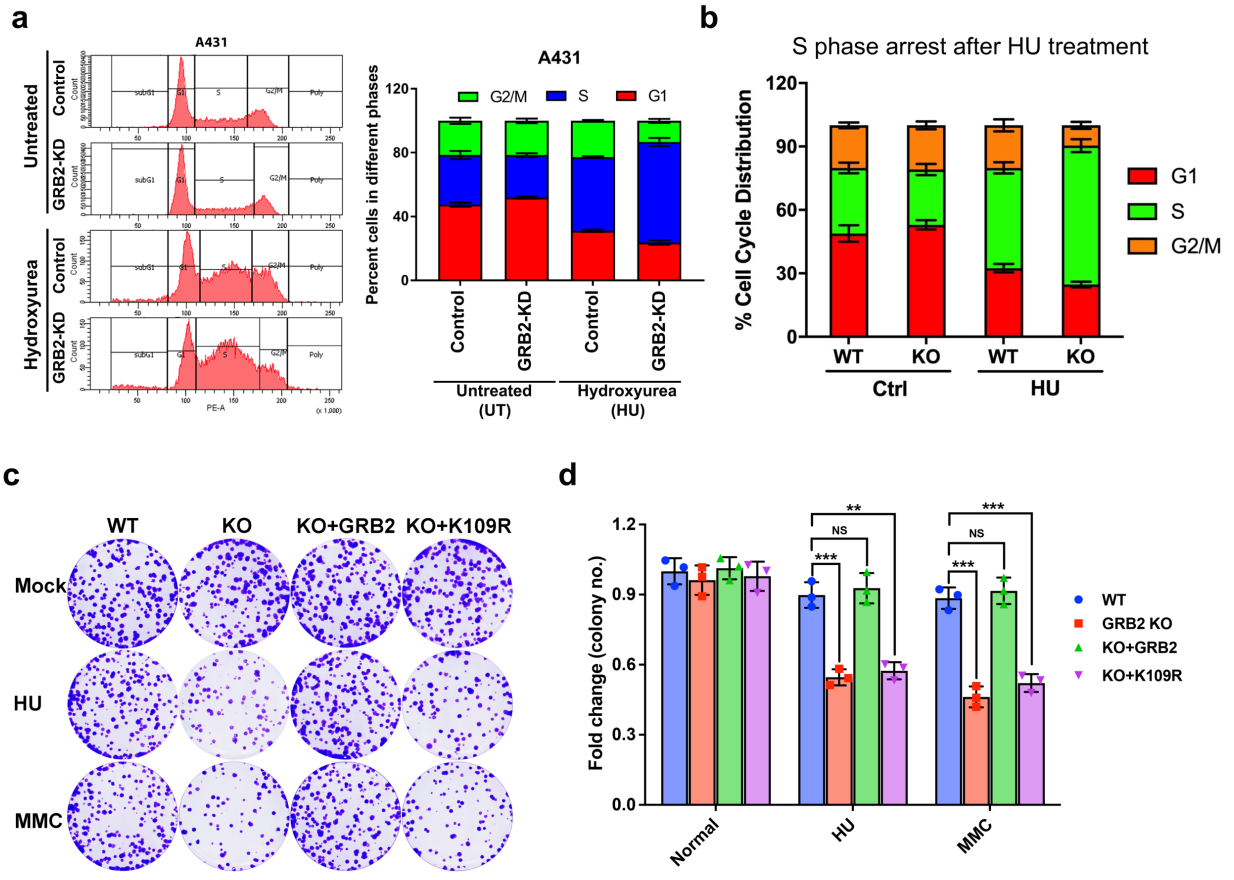

Supplementary Fig. 1: GRB2 depleted cells are sensitive to DNA replication stress.
a, Representative raw cell-cycle analysis data of propidium iodide stained A431 cells containing a scrambled shRNA (Control) or GRB2 shRNA (GRB2-KD). Cells were either left untreated or treated with 0.5mM hydroxyurea (HU) for 24h. Quantitation are shown on the right. **b,** Quantitation of control (Ctrl) and GRB2-KO HeLa cells at different stage of cell-cycle following 24h 0.5mM hydroxyurea treatment. Both GRB2-KD A431 and GRB2-KO HeLa cells showing higher percentage of cell stagnation at S-phase following HU treatment. **c,** Colony survival assay of HeLa cells treated with a single dose 0.5mM HU or 0.1mM mitomycin C. 10 day after treatment cells were stained with trypan blue imaged with densitometer. **d,** Quantitation of colony survival assay data from three independent

experiments. The significance was analyzed by two-sided Student's t test. ***$P \leq 0.001$ and NS, not significant. Error bars showing standard deviations (SD).

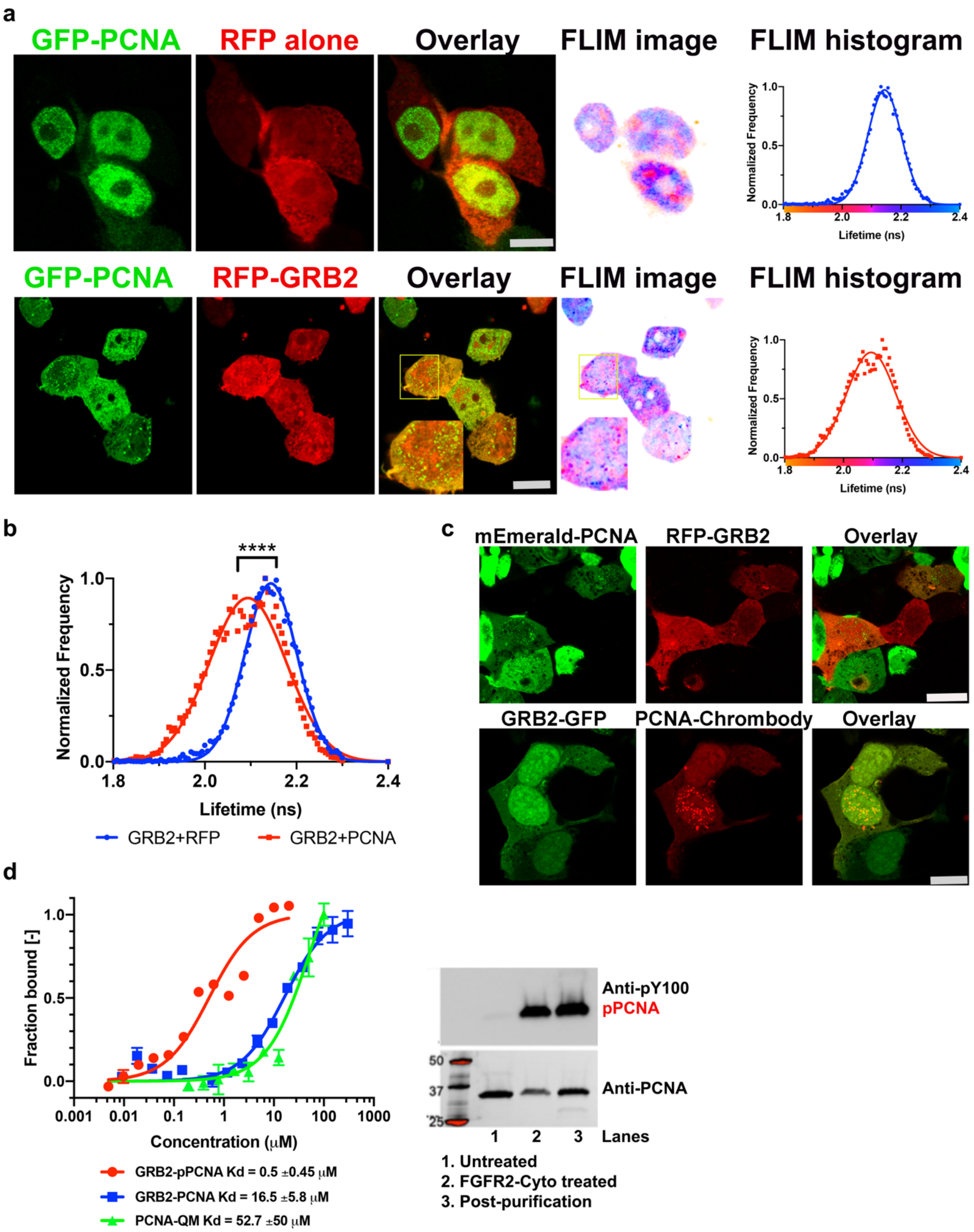

Supplementary Fig. 2: Measurements of GRB2-PCNA direct interactions.

a, Fluorescence lifetime imaging microscopy (FLIM) (1) measuring fluorescence resonance energy transfer (FRET) from GFP-PCNA to RFP-GRB2. Individual along with colocalized images are shown.

The lifetime image shown as a false color image generated by pixel-by-pixel mapping of the measured lifetime-values represented by the scale 1.8-2.4 nanosecond (ns). The FLIM histograms showing normalized frequency distribution of lifetime for the field of view image. Scale bar 25μm. **b,** Overlay of the two FLIM histogram to demonstrate a left-shifted distribution of lifetime for sample containing donor (GFP-PCNA) and acceptor (RFP-GRB2) as binding partner. Raw data was fitted in Prism 8 with Gaussian fitting parameter. Two-way ANOVA test, $P = 0.0001$. **c,** Confocal imaging of PCNA overexpression. Co-localization of monomeric Emerald (mEmerald) tagged PCNA without the nuclear localization (NLS) sequence and n-terminal RFP-tagged GRB2 (RFP-GRB2) showing diffused distributions with nuclear PCNA-foci (top panels). Co-overexpression of TagRFP-tagged PCNA chromobody and c-terminal GFP-tagged GRB2. Scale bar 25μm. **d,** MST binding isotherms measuring 100nM Atto488 labelled GRB2 interaction with titrating concentration of PCNA (GRB2-PCNA). 100nM Atto488 labelled PCNA mixed with titrating concentration of GASHGQTGMFPRNYVT peptide. the resulting binding isotherm and Kd (μM) is shown (PCNA-QM). Also shown titrating concentration of tyrosine phosphorylated PCNA interaction with fixed 100nM Atto488 labelled GRB2 (GRB2-pPCNA). **e,** Western analysis for the tyrosine phosphorylated PCNA used for MST binding studies in '**c**'. Bacterially purified 100nM FGFR2 cytoplasmic domain as described previously (2) and 1mg PCNA was mixed in the presence of ATP/Mg^{2+} for 4 hours then run through an SEC column. Phosphorylated PCNA fraction then isolated using Phosphoprotein purification column (Takara).

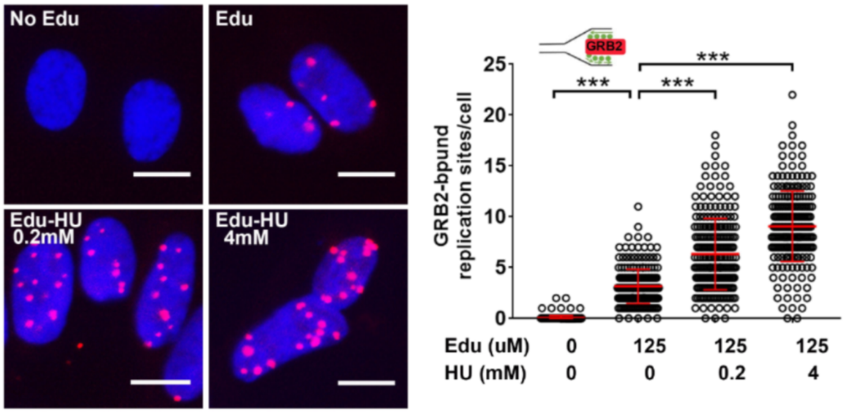

Supplementary Fig. 3: In situ analysis of protein interactions at DNA replication forks (SIRF) assay (3) showing hydroxyurea (HU) mediated replication stress induced GRB2 accumulation at replication sites. The significance was analyzed by two-sided Student's t-test. ***P ≤ 0.001 and NS, not significant. Error bars showing standard deviations (SD). Scale bar 10μm.

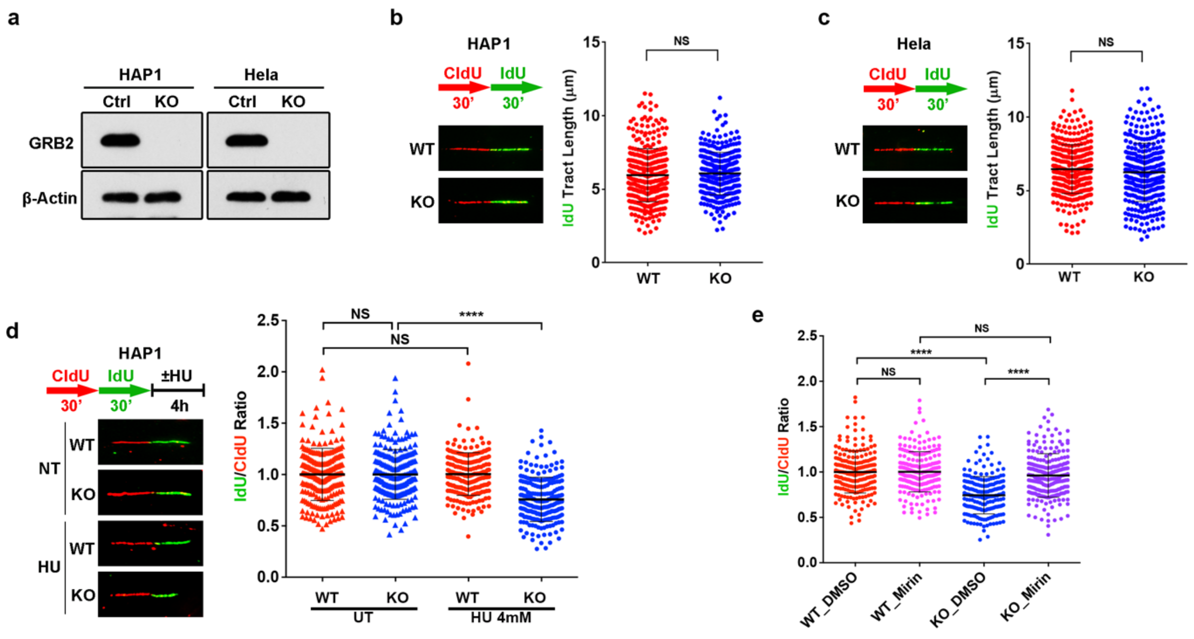

Supplementary Fig. 4: Replication forks of GRB2-depleted cells are susceptible to MRE11 dependent degradation.

a, Western blot analysis of total cellular protein from control and GRB2 knockout HeLa and HAP-1 cells. Anti-beta actin antibody was used as loading control. **b-c,** DNA fiber assay showing a comparison of DNA replication fork between control and GRB2 KO HeLa and HAP-1 cells under normal media growth condition. **d,** DNA fiber assay showing enhanced fork degradation in GRB2-depleted HAP-1 cells under replication stress. **e,** Fork degradation is inhibited by MRE11 nuclease inhibitor Mirin in HAP1 cells lacking GRB2 and under replication stress. (The significance was analyzed by two-sided Student's t test. ***P ≤ 0.001, and ****P ≤ 0.0001; NS, not significant.

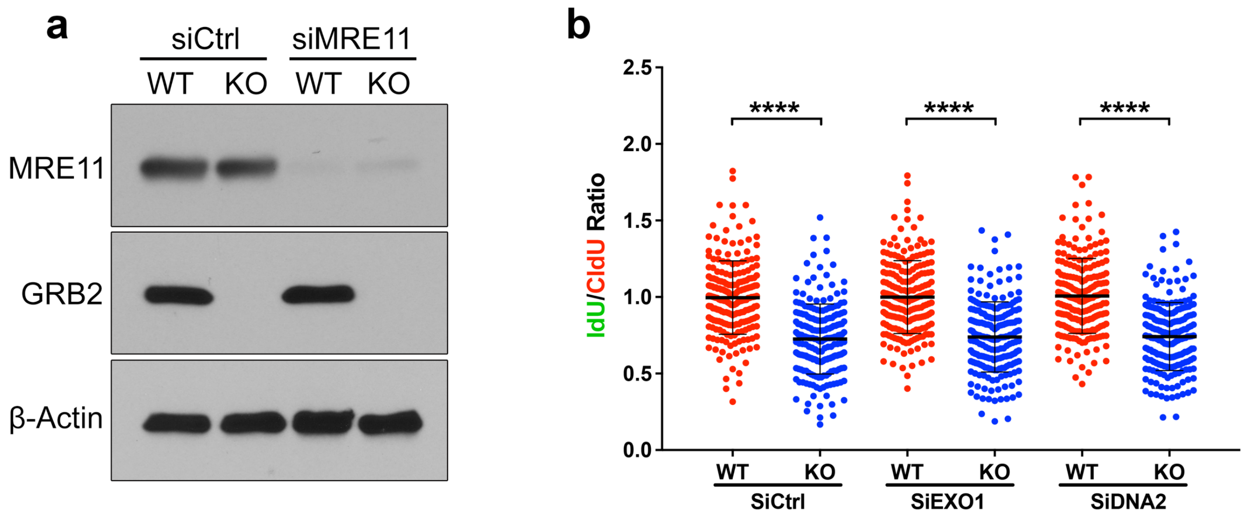

Supplementary Fig. 5: MRE11 specifically degrade RFs in GRB2 depleted cells. a, western analysis of MRE11-knockdown (siMRE11) in control and GRB2-KO HeLa cells. **b,** The knockdown of EXO1 or DNA2 failed to prevent fork degradation in GRB2-deplered HeLa cells under replication stress. The significance was analyzed by two-sided Student's t test. ***P ≤ 0.001.

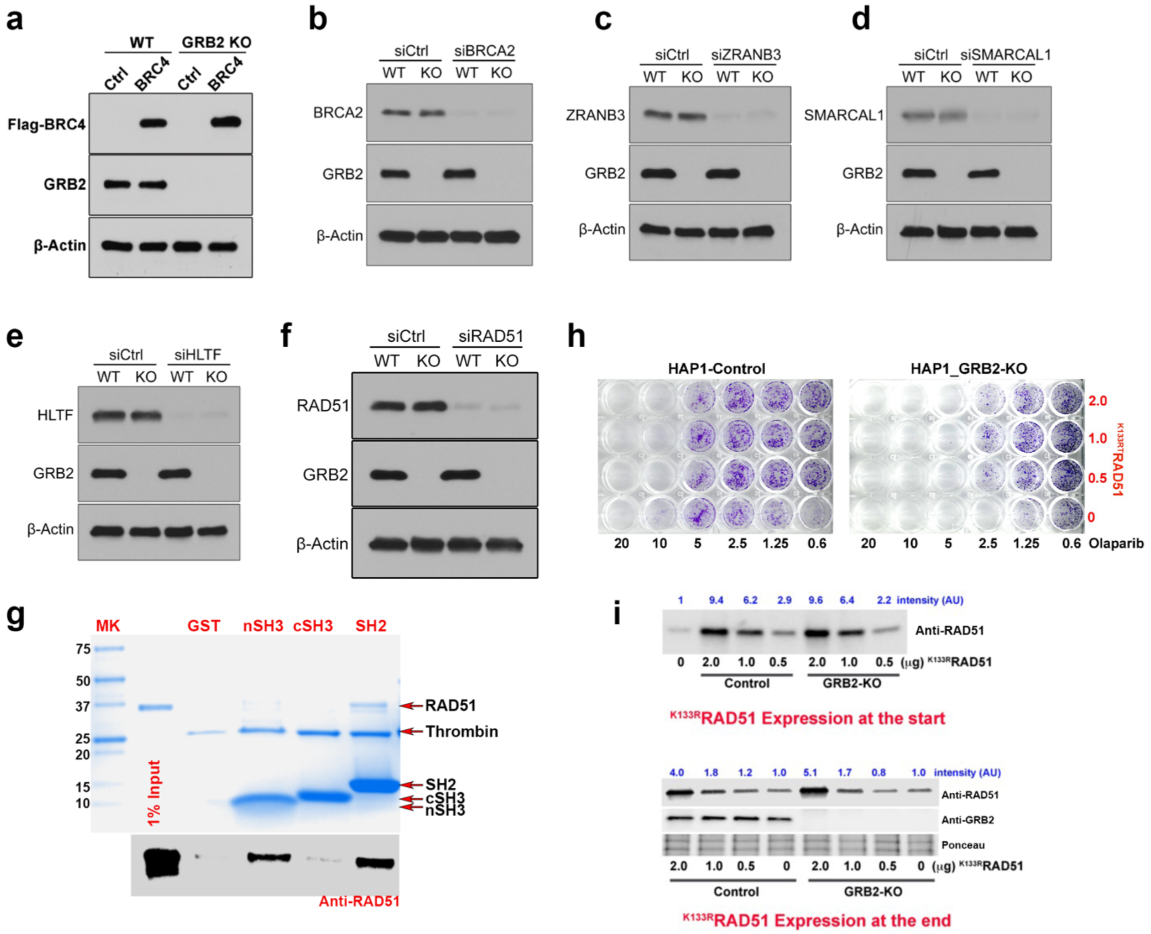

Supplementary Fig. 6: Western analysis for protein knockdowns plus the functional effect of ^{K133R}RAD51 expression on PARPi mediated GRB2-KO cell-killing.

a-f, Western blotting analysis validating siRNA mediated knockdown of the designated proteins with the indicated antibody. Anti-GRB2 and anti-beta actin was also used to confirm GRB2-KO and loading control respectively.

g, An *in vitro* pulldown assay and Coomassie blue detection showing GRB2-SH2 domain specifically binds RAD51. Thrombin cleavage was used to elute SH-domain from the GSH agarose beads. The position of RAD51, thrombin and the individual SH-domains are shown with arrows. A parallel gel was immunoblotted with anti-RAD51 antibody confirming GRB2-SH2 domain as the primary interaction site. The sensitivity of western analysis also suggests the nSH3 domain specifically is also capable of making a contact with RAD51. **h,** Colony survival PARPi dose-response assay of control and GRB2-KO cells transfected with increasing concentrations of K133R mutant RAD51 (^{K133R}RAD51). Cells were treated with indicated concentration of Olaparib for 8 consecutive days before visualization with trypan blue staining. **i,** Western analysis of ^{K133R}RAD51 expression at the start (top panel) and on the final day-8 of the clonogenic assay (bottom panels). The relative expression quantification of intensity adjusted to un-transfected (0) are shown as arbitrary unit (AU).

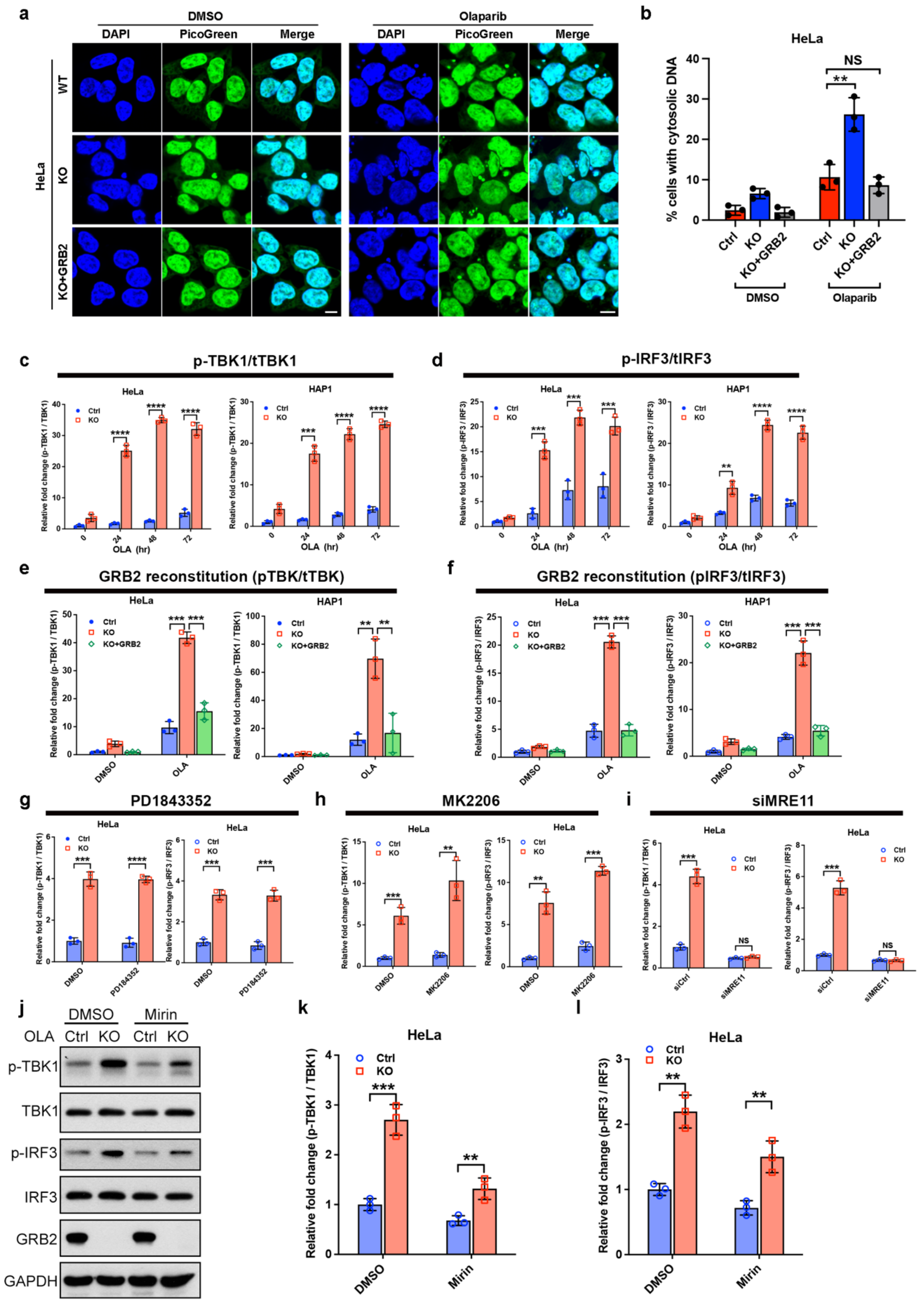
a
DMSO
Olaparib
DAPI
PicoGreen
Merge
HeLa
WT
KO
KO+GRB2
b
HeLa
% cells with cytosolic DNA
NS
**
Ctrl
KO
KO+GRB2
DMSO
Olaparib
c
p-TBK1/tTBK1
HeLa
HAP1
Relative fold change (p-TBK1 / TBK1)
OLA (hr)
d
p-IRF3/tIRF3
Relative fold change (p-IRF3 / IRF3)
e
GRB2 reconstitution (pTBK/tTBK)
f
GRB2 reconstitution (pIRF3/tIRF3)
g
PD1843352
PD184352
h
MK2206
i
siMRE11
siCtrl
j
OLA
Mirin
p-TBK1
TBK1
p-IRF3
IRF3
GRB2
GAPDH
k
l

Supplementary Fig. 7: PARPi induced micronuclei formation in GRB2 depleted HeLa cells. a, Representative PicoGreen and DAPI staining showing Olaparib-induced cytoplasmic micronuclei formation in HeLa cells. Scale bar 10μm. **b,** Quantitation of cytoplasmic micronuclei form three independent experiments are shown in the left panel. **c-i,** Quantitation of pTBK1 and IRF3 levels in three independent western blotting data from HeLa and HAP1 cells. **j-i,** The effect of MRE11 inhibitors Mirin on pTBK1 level in control and GRB2-KO cells. HeLa cells treated with 10μM Olaparib and with or without 10μM Mirin for 48h. Total cell lysates were immunoblotted for the indicated antibody. **j**; representative western blot, **k**; quantification of pTBK1/totalTBK1 from three independent experiments, **l**; quantification of pIRF3/totalIRF3 from three independent experiments. The significance was analyzed by two-sided Student's t test. **P ≤ 0.01; ***P ≤ 0.001; ****P ≤ 0.0001; NS, not significant.

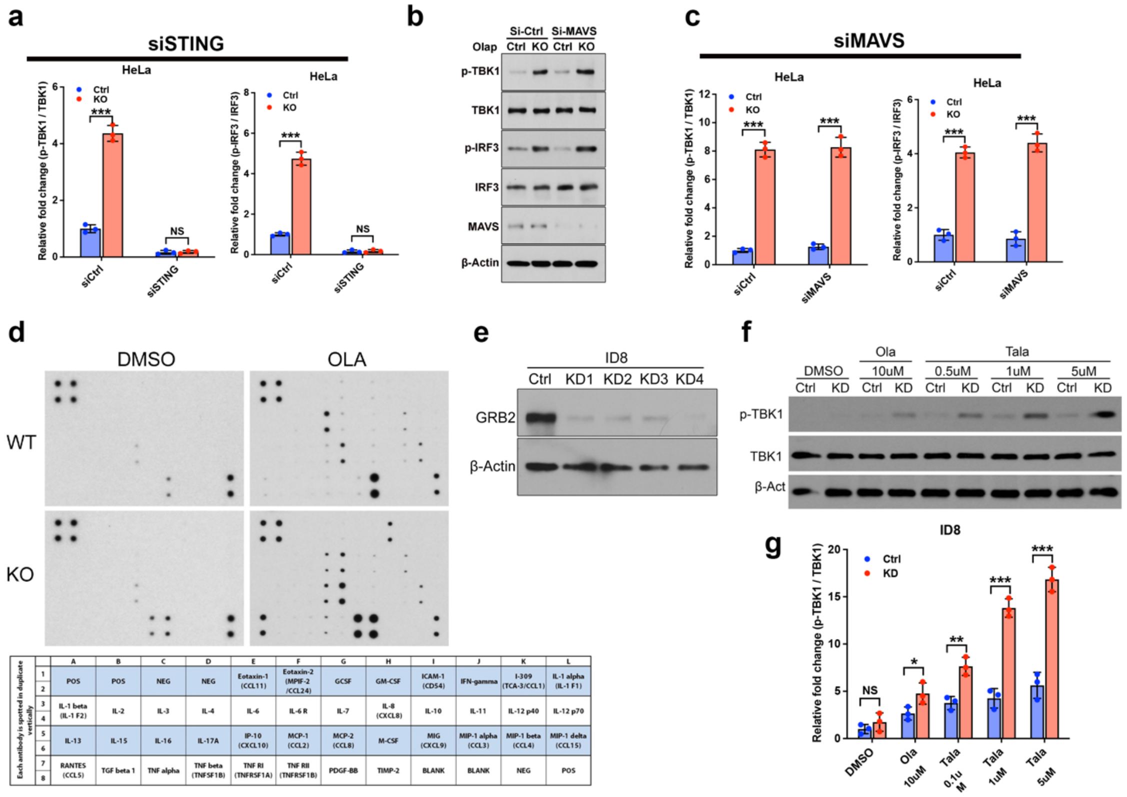

Each antibody is spotted in duplicate vertically		A	B	C	D	E	F	G	H	I	J	K	L
	1, 2	POS	POS	NEG	NEG	Eotaxin-1 (CCL11)	Eotaxin-2 (MPIF-2 /CCL24)	GCSF	GM-CSF	ICAM-1 (CD54)	IFN-gamma	I-309 (TCA-3/CCL1)	IL-1 alpha (IL-1 F1)
	3, 4	IL-1 beta (IL-1 F2)	IL-2	IL-3	IL-4	IL-6	IL-6 R	IL-7	IL-8 (CXCL8)	IL-10	IL-11	IL-12 p40	IL-12 p70
	5, 6	IL-13	IL-15	IL-16	IL-17A	IP-10 (CXCL10)	MCP-1 (CCL2)	MCP-2 (CCL8)	M-CSF	MIG (CXCL9)	MIP-1 alpha (CCL3)	MIP-1 beta (CCL4)	MIP-1 delta (CCL15)
	7, 8	RANTES (CCL5)	TGF beta 1	TNF alpha	TNF beta (TNFSF1B)	TNF RI (TNFRSF1A)	TNF RII (TNFRSF1B)	PDGF-BB	TIMP-2	BLANK	BLANK	NEG	POS

Supplementary Fig. 8: MAVS pathway is not involved in GRB2-KO cells.

a, Quantification of pTBK1 and pIRF3 phosphorylation levels in the control and GRB2-KO HeLa cells. Data from three independent experiments are shown. **b,** representative western blot of the effect of Olaparib treatment (10μM for 48h) on TBK-1 and IRF-3 phosphorylation in GRB2-KO HeLa cells depleted of MAVS. MAVS was knocked down in control and GRB2-KO HeLa cells with siRNA, and olaparib induced pTBK1 and pIRF3 were measured. **c,** western blot quantification from three independent experiments. ***P ≤ 0.001; NS, not significant. **d,** Representative image of a cytokine array slide. Control and GRB2-KO HeLa cells untreated or treated with 10 μM Olaparib for 48h then the supernatants were analyzed for pro-inflammatory cytokines in cell-media. Tested cytokines with their relative grid positions are shown below. **e,** Knockdown (KD) efficiency of mouse Ovarian cancer cells ID8 evaluated with anti-GRB2 antibody and anti-beta actin as loading control. GRB2 expression level in KD clones 1-4 are shown. The clone KD4 showed the most GRB2 depletion. **f,** Testing of PARPi Olaparib and Talazoparib responses by mouse ID8 clone-KD4. Anti-pTBK1 antibody was used to evaluate cGAS/STING activation. Total TBK1 and beta actin were used as loading controls. Cells were treated for 48h by the indicated inhibitor concentration of PARPi **g,** Quantification of Olaparib induced pTBK1 level in ID8 cells from three independent experiments. The significance was analyzed by two-sided Student's t test. **P ≤ 0.01; ***P ≤ 0.001; ****P ≤ 0.0001; NS, not significant.

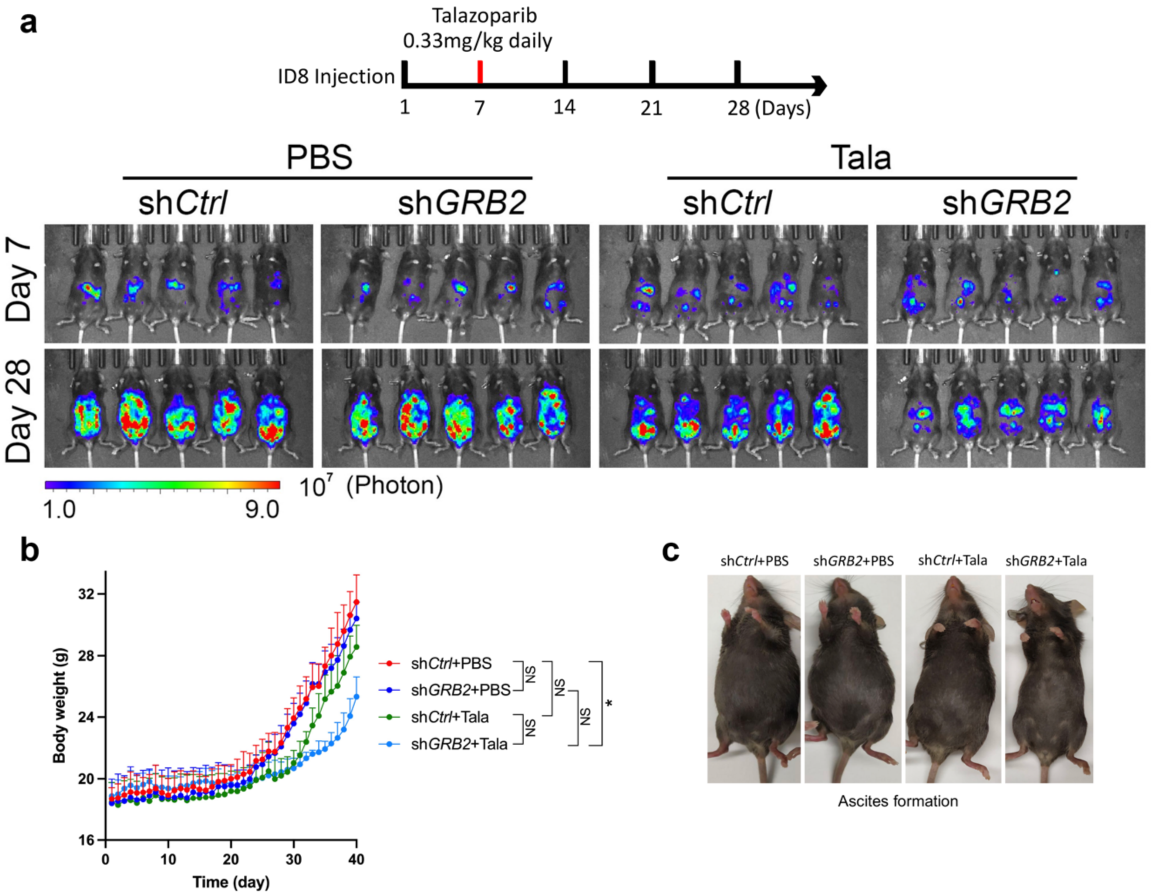

Supplementary Fig. 9*:* Luciferase imaging and ascites.

a, in vivo mouse study to evaluate the effect of PARPi treatment in cells expressing low-level GRB2. Luciferase imaging of mice at day 7 and day 28 are shown. **b,** the body weight measurements of all treated animals. **c,** representative images of mouse with apparent high body weight due to increased ascites fluid accumulation.

Supplementary References:

1. Ahmed Z, Timsah Z, Suen KM, Cook NP, Lee GRt, Lin CC, Gagea M, Marti AA, Ladbury JE. Grb2 monomer-dimer equilibrium determines normal versus oncogenic function. Nat Commun. 2015;6:7354. Epub 2015/06/25. doi: 10.1038/ncomms8354. PubMed PMID: 26103942; PMCID: PMC4491180.

2. Lin CC, Melo FA, Ghosh R, Suen KM, Stagg LJ, Kirkpatrick J, Arold ST, Ahmed Z, Ladbury JE. Inhibition of basal FGF receptor signaling by dimeric Grb2. Cell. 2012;149(7):1514-24. doi: 10.1016/j.cell.2012.04.033. PubMed PMID: 22726438.

3. Roy S, Luzwick JW, Schlacher K. SIRF: Quantitative in situ analysis of protein interactions at DNA replication forks. J Cell Biol. 2018;217(4):1521-36. Epub 2018/02/25. doi: 10.1083/jcb.201709121. PubMed PMID: 29475976; PMCID: PMC5881507.
