# Supplementary material for: GRB2 stabilizes RAD51 at reversed replication forks suppressing genomic instability and innate immunity against cancer
Source: Nat Commun. 2024 Mar 8;15:2132. doi: 10.1038/s41467-024-46283-y (PMC10923831; doi:10.1038/s41467-024-46283-y)
Supplement: Supplementary file 3 — Reporting Summary [file 41467_2024_46283_MOESM3_ESM.pdf]

nature portfolio

Corresponding author(s): Zamal Ahmed

Last updated by author(s): Jan 23, 2024

Reporting Summary

Nature Portfolio wishes to improve the reproducibility of the work that we publish. This form provides structure for consistency and transparency in reporting. For further information on Nature Portfolio policies, see our Editorial Policies and the Editorial Policy Checklist.

Statistics

For all statistical analyses, confirm that the following items are present in the figure legend, table legend, main text, or Methods section.

n/a	Confirmed	
☐	☒	The exact sample size (*n*) for each experimental group/condition, given as a discrete number and unit of measurement
☐	☒	A statement on whether measurements were taken from distinct samples or whether the same sample was measured repeatedly
☐	☒	The statistical test(s) used AND whether they are one- or two-sided<br>*Only common tests should be described solely by name; describe more complex techniques in the Methods section.*
☐	☒	A description of all covariates tested
☐	☒	A description of any assumptions or corrections, such as tests of normality and adjustment for multiple comparisons
☐	☒	A full description of the statistical parameters including central tendency (e.g. means) or other basic estimates (e.g. regression coefficient) AND variation (e.g. standard deviation) or associated estimates of uncertainty (e.g. confidence intervals)
☐	☒	For null hypothesis testing, the test statistic (e.g. *F*, *t*, *r*) with confidence intervals, effect sizes, degrees of freedom and *P* value noted<br>*Give P values as exact values whenever suitable.*
☒	☐	For Bayesian analysis, information on the choice of priors and Markov chain Monte Carlo settings
☒	☐	For hierarchical and complex designs, identification of the appropriate level for tests and full reporting of outcomes
☒	☐	Estimates of effect sizes (e.g. Cohen's *d*, Pearson's *r*), indicating how they were calculated

Our web collection on statistics for biologists contains articles on many of the points above.

Software and code

Policy information about availability of computer code

Data collection	No software was used for data collection.
Data analysis	GraphPad Prism (version 8.0)FlowJo (version 10.7.1)

For manuscripts utilizing custom algorithms or software that are central to the research but not yet described in published literature, software must be made available to editors and reviewers. We strongly encourage code deposition in a community repository (e.g. GitHub). See the Nature Portfolio guidelines for submitting code & software for further information.

Data

Policy information about availability of data

All manuscripts must include a data availability statement. This statement should provide the following information, where applicable:

- Accession codes, unique identifiers, or web links for publicly available datasets
- A description of any restrictions on data availability
- For clinical datasets or third party data, please ensure that the statement adheres to our policy

The authors declare that all the supporting data of this study are included within the manuscript. All the Supplementary Information files can be obtained from the authors. Source data are provided with this paper.

Research involving human participants, their data, or biological material

Policy information about studies with human participants or human data. See also policy information about sex, gender (identity/presentation), and sexual orientation and race, ethnicity and racism.

Reporting on sex and gender	Only human blood was used in this study and it was provided by MD Anderson blood center. Sex is not considered in the study design and the donors' information is not provided by MD Anderson blood center.
Reporting on race, ethnicity, or other socially relevant groupings	Only human blood was used in this study and it was provided by MD Anderson blood center. Race, ethnicity, or other socially relevant groupings is not considered in the study design and the all the donors' information are not provided by MD Anderson blood center.
Population characteristics	Only human blood was used in this study and it was provided by MD Anderson blood center. Population characteristics is not considered in the study design and not provided by MD Anderson blood center.
Recruitment	We obtained human blood from MD Anderson blood center and the project did not involve patient recruitment
Ethics oversight	The Research Ethics Committees of MD Anderson Cancer Center

Note that full information on the approval of the study protocol must also be provided in the manuscript.

Field-specific reporting

Please select the one below that is the best fit for your research. If you are not sure, read the appropriate sections before making your selection.

☒ Life sciences ☐ Behavioural & social sciences ☐ Ecological, evolutionary & environmental sciences

For a reference copy of the document with all sections, see nature.com/documents/nr-reporting-summary-flat.pdf

Life sciences study design

All studies must disclose on these points even when the disclosure is negative.

Sample size	Group sizes for in vitro experiments were selected on the basis of prior knowledge of variation. Group sizes for in vivo experiments were based upon prior knowledge of the individual variation within mice, in which group sizes of 5-7 mice treatment is acceptable (PMID: 34671158, PMID: 35165416 and PMID: 32581358).
Data exclusions	No data was excluded during the statistical process.
Replication	All experiments were repeated at least three times independently, with all repetitions yielding consistent results.
Randomization	All the animals were allocated into experiment groups randomly.
Blinding	The investigator were blinded during data analysis process.

Reporting for specific materials, systems and methods

We require information from authors about some types of materials, experimental systems and methods used in many studies. Here, indicate whether each material, system or method listed is relevant to your study. If you are not sure if a list item applies to your research, read the appropriate section before selecting a response.

Materials & experimental systems

n/a	Involved in the study	
☐	☒	Antibodies
☐	☒	Eukaryotic cell lines
☒	☐	Palaeontology and archaeology
☐	☒	Animals and other organisms
☒	☐	Clinical data
☒	☐	Dual use research of concern
☒	☐	Plants

Methods

n/a	Involved in the study	
☒	☐	ChIP-seq
☐	☒	Flow cytometry
☒	☐	MRI-based neuroimaging

Antibodies

Antibodies used	Anti-PCNA (2586), Anti-STING (13647), Anti-MAVS (3993) anti-cGAS (15102), Anti-ERK1/2 (4370), Anti-ERK1/2 (4695), Anti-p-Akt

Antibodies used	(4060), Anti-Akt (4685), anti-IRF3 (11904), anti-p-IRF3 (29047), anti-TBK1 (3504), anti-p-TBK1 (5483), anti-MRE11 (4847), anti-Histone H3 (4499) and anti-b-Actin (3700), Anti-Flag-tag (14793), Anti-GAPDH (2118), BRCA2 (10741), SMARCAL1(44717), HTLF (43345) antibodies were ordered from Cell Signaling Technology. Anti-GRB2 (C-23) (sc-255), was purchased from Santa Cruz Biotechnology. Anti-MRE11 (ab214) were purchased from abcam. Anti-BrdU (347580) was from BD Biosciences. Anti-Rad51 (GTX100469) and ZRANB3 (GTX66576) were ordered from GeneTex. CD8 (301014), Mouse IL2, Mouse IL-12 p40 (ELISA kit 431004) were from Biolegend Olaparib, MK2206, PD184352 and BMN673 (S7048, Talazoparib) was ordered from Selleck Chemicals.
Validation	Only commercially available antibodies were used. According to manufacturer's website, all the antibodies were validated for the usages of species and application in this manuscript.

Eukaryotic cell lines

Policy information about cell lines and Sex and Gender in Research

Cell line source(s)	HeLa cells were ordered from ATCC , and HAP1 cells were purchased from Horizon Discovery. The ID8 mouse ovarian cancer cells were kindly provided by Dr. Guang Peng's laboratory (MD Anderson Cancer Center).
Authentication	All cell lines used in this study have been authenticated by STR before the start of experiments.
Mycoplasma contamination	All the cell lines used in this study were tested negative for mycoplasma contamination.
Commonly misidentified lines (See ICLAC register)	No commonly misidentified lines were used in this study.

Animals and other research organisms

Policy information about studies involving animals; ARRIVE guidelines recommended for reporting animal research, and Sex and Gender in Research

Laboratory animals	6-8 week old female BC57BL/6 mice were purchased from Hangzhou Medical College. All mice were housed in pathogen-free facilities (SPF) in the MD Anderson Cancer Center animal facilities. All mice were fed with filtered water and sterilized food under standard conditions, at 72 degrees Fahrenheit, 65% humidity, 12 hour light/ 12 hour dark cycles.
Wild animals	We did not use any wild animals.
Reporting on sex	We used the mouse ovarian cancer cell line ID8 to establish the animal model. Therefore, only female mouse were included in this study.
Field-collected samples	No field-collected samples were used in this study.
Ethics oversight	All the animal experimental procedures were guided and approved by MD Anderson's Institutional Animal Care and Use Committee.

Note that full information on the approval of the study protocol must also be provided in the manuscript.

Flow Cytometry

Plots

Confirm that:

☒ The axis labels state the marker and fluorochrome used (e.g. CD4-FITC).

☒ The axis scales are clearly visible. Include numbers along axes only for bottom left plot of group (a 'group' is an analysis of identical markers).

☒ All plots are contour plots with outliers or pseudocolor plots.

☒ A numerical value for number of cells or percentage (with statistics) is provided.

Methodology

Sample preparation	Human blood was provided by MD Anderson blood center. Peripheral blood mononuclear cells (PBMCs) were purified with Ficoll (SigmaAldrich) by density gradient separation. WT and GRB2 KO HeLa cells were pretreated with 20 µM Olaparib for 48 hours were seeded onto 24-well plates, and PBMCs were then added on 3-µm hanging cell culture inserts (Millicell, No. MCMP24H48) placed in a 24-well plate. After overnight incubation, cells that migrated to the 24 wells were collected, counted, and stained with fixable viability dye (Invitrogen, No. 65-0863-14) followed by APC/Cyanine7 anti-mouse CD45, FITC anti-CD3, PerCP/Cyanine5.5 anti-CD8 antibodies.
Instrument	Samples were analyzed by FACS Canto II flow cytometer.
Software	FlowJo (version 10.7.1)
Cell population abundance	No cell sorting was performed in this manuscript.

Gating strategy

All gates were set based on cell lines and isotype control antibodies after appropriate compensation using singles tained compensation controls.

☒ Tick this box to confirm that a figure exemplifying the gating strategy is provided in the Supplementary Information.
